# Primary hyperparathyroidism in pregnancy successfully treated with microwave ablation: a case report

**DOI:** 10.3389/fmed.2025.1688249

**Published:** 2026-01-23

**Authors:** Abderrahim Bey

**Affiliations:** Cabinet Médical Docteur Bey Abderrahim, Algiers, Algeria

**Keywords:** hypercalcemia, microwave ablation (MW ablation), parathyroid adenoma, pregnancy, primary hyperparathyroidism (pHPT)

## Abstract

**Background:**

Primary hyperparathyroidism (PHPT) during pregnancy is rare, with an estimated prevalence of <1% among pregnant women. While often asymptomatic, it may lead to serious maternal complications (nephrolithiasis, pancreatitis, preeclampsia) and adverse fetal outcomes (growth restriction, miscarriage, preterm delivery, stillbirth). Surgical parathyroidectomy in the second trimester remains the gold standard treatment, but may be unfeasible due to gestational age, contraindications, or patient refusal. In such situations, minimally invasive techniques such as microwave ablation (MWA) have emerged as potential alternatives, though their use in pregnancy is extremely rare, with only one case previously reported.

**Case:**

We report the case of a 38-year-old woman in her second trimester, with a history of multiple miscarriages and no living children, referred for hypercalcemia discovered on routine screening. Laboratory tests revealed corrected serum calcium of 11.54 mg/dL (85–105), elevated intact parathyroid hormone (iPTH) at 100.3 pg./mL (15–65), and marked hypercalciuria. Cervical ultrasound demonstrated a vascularized hypoechoic lesion posterior to the left thyroid lobe, consistent with a parathyroid adenoma, further confirmed by fine-needle aspiration with PTH washout (2075 pg./mL). After refusal of surgery, ultrasound-guided MWA was performed under local anesthesia with hydrodissection to protect adjacent structures.

**Results:**

Within 1 h, iPTH dropped to 16.8 pg./mL and serum calcium normalized to 10.0 mg/dL, remaining stable throughout pregnancy. Symptoms resolved within days. Obstetric monitoring confirmed healthy fetal development. At 38 weeks, the patient delivered a healthy infant by cesarean section. No maternal or fetal complications occurred.

**Conclusion:**

This case represents only the second reported use of MWA for PHPT during pregnancy, demonstrating its feasibility, efficacy, and safety in a highly selected patient. MWA may provide a valuable minimally invasive alternative when surgery is contraindicated or refused, though further evidence is needed to define its role in this unique clinical context.

## Introduction

Primary hyperparathyroidism (PHPT) during pregnancy is an uncommon endocrine disorder, with an estimated prevalence of less than 1% among pregnant women (1, 2). Although often asymptomatic, it can be associated with significant maternal complications, including nephrolithiasis, pancreatitis, and preeclampsia, as well as adverse fetal outcomes such as intrauterine growth restriction, preterm delivery, and stillbirth (3–6). The condition is most frequently caused by a single parathyroid adenoma, with multigland disease and parathyroid carcinoma being rare in this population (7).

Surgical removal of the adenoma, preferably during the second trimester, remains the gold standard treatment for symptomatic or severe disease, as it is the only curative option (8). In some urgent cases, such as acute pancreatitis related to primary hyperparathyroidism, surgery may be required outside this optimal period (9). However, surgery may not be feasible in certain clinical scenarios, including advanced gestational age, contraindications to general anesthesia, or patient refusal (10). In such situations, medical management can be attempted but is often limited by efficacy and safety concerns (11).

In recent years, minimally invasive image-guided thermal ablation techniques, including radiofrequency ablation (RFA) and microwave ablation (MWA), have emerged as potential alternatives to surgery for selected patients with PHPT (12–14), though their use during pregnancy remains extremely rare and poorly documented (15). We report the case of a 38-year-old pregnant woman with symptomatic PHPT successfully treated with ultrasound-guided MWA of a parathyroid adenoma, highlighting the potential role of this technique in carefully selected cases.

This case is also notable given the patient’s advanced maternal age and history of recurrent miscarriages, making the pregnancy especially precious and the therapeutic decision particularly delicate.

## Case presentation

A 38-year-old pregnant woman in her second trimester was referred by her gynecologist for evaluation of hypercalcemia discovered incidentally during routine blood tests. She had a history of multiple unexplained miscarriages and no living children. Her presenting symptoms included persistent fatigue, exaggerated pregnancy-related nausea and vomiting, and poor response to symptomatic treatment.

Initial laboratory evaluation revealed a markedly elevated corrected serum calcium level of 115.4 mg/L (2.89 mmol/L) (reference range: 85–105 mg/L [2.12–2.62 mmol/L]), an inappropriately high parathyroid hormone (iPTH) level of 100.3 pg./mL (15–65), and marked hypercalciuria at 558 mg/24 h, with a vitamin D level at the lower end of the normal range. She also had a history of Hashimoto’s thyroiditis, for which she was receiving levothyroxine replacement therapy.

**Figure 1:**
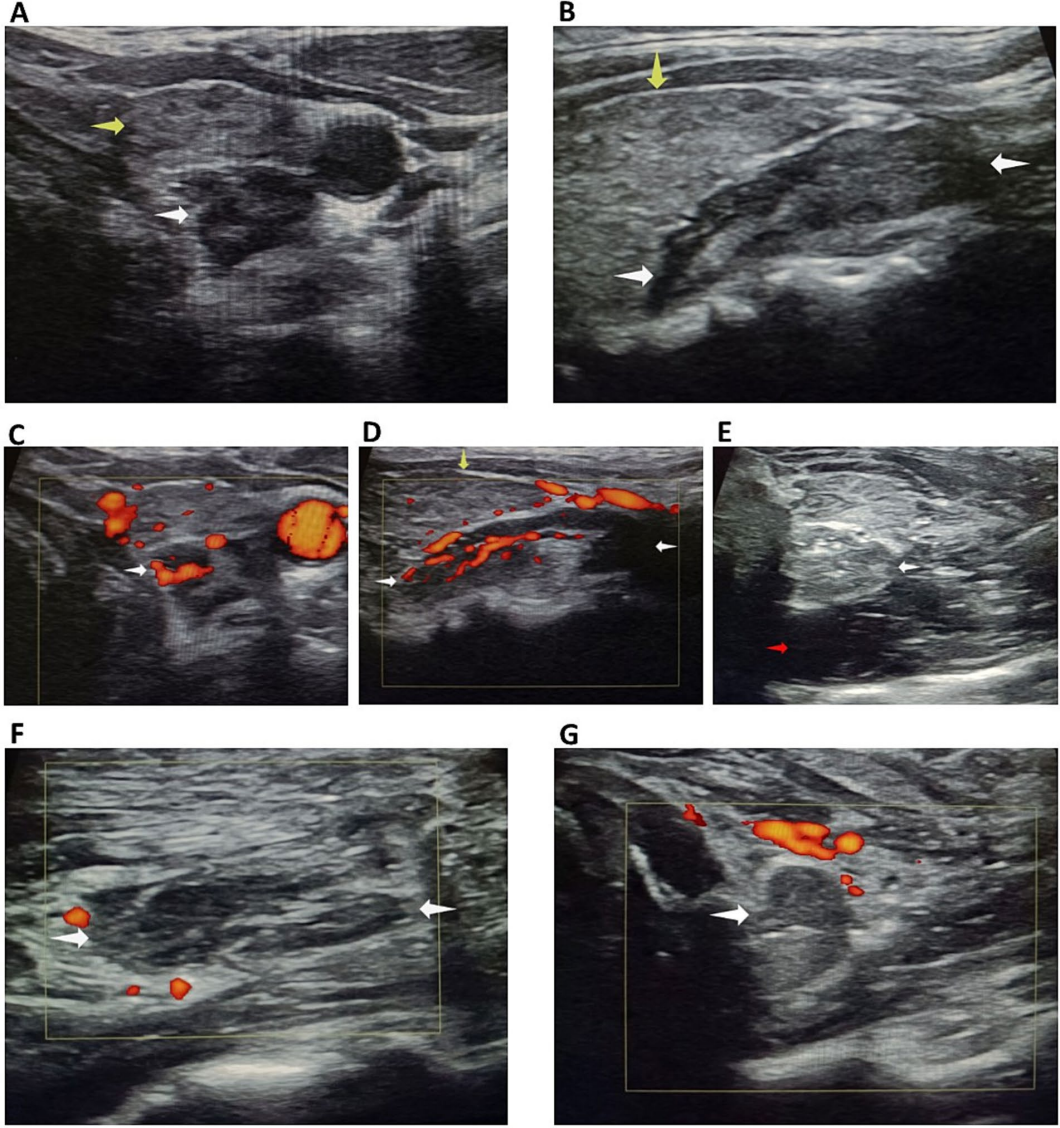
**(A,B)** Large hypoechoic oblong lesion at the posterior aspect of the upper pole of the left thyroid lobe. **(C,D)** Clearly identifiable feeding artery. **(E)** Posterior hydrodissection with glucose serum. **(F,G)** Pronounced hypoechogenicity with complete absence of vascularization at the end of the procedure. White arrow: parathyroid adenoma; yellow arrow: thyroiditis; red arrow: glucose serum.

Cervical ultrasound demonstrated a large hypoechoic, oblong lesion located posterior to the left superior thyroid lobe, measuring 26.1 × 13.3 × 10.1 mm (volume: 1.82 mL) (Figures 1A,B). The lesion was highly vascularized, with a clearly identifiable feeding artery (Figures 1C,D), strongly suggestive of a parathyroid adenoma. Although sestamibi scintigraphy can exceptionally be performed with reduced radioisotope doses in selected cases, we deliberately avoided any radiation exposure during pregnancy. Instead, the parathyroid origin of the lesion was reliably confirmed by fine-needle aspiration with *in situ* PTH washout, yielding a markedly elevated PTH concentration of 2075 pg./mL, thus providing a non-radiative and highly specific diagnostic confirmation.

Due to the unavailability of genetic testing in our region, multiple endocrine neoplasia (MEN) could not be formally excluded at the genetic level. Therefore, MEN was assessed through a thorough clinical and biochemical evaluation. A detailed family history and clinical assessment revealed no findings suggestive of MEN. Biochemical testing included serum calcitonin measurement, methoxylated derivatives, and a comprehensive pituitary hormonal profile, all of which were within normal ranges. Nevertheless, the absence of genetic testing represents a limitation of this case.

In the absence of a clearly identified cause of recurrent miscarriages, primary hyperparathyroidism could be considered a possible contributing factor, although a formal causal relationship cannot be established.

### Management

Initial treatment included intravenous hydration, which slightly reduced calcium levels to 112–113 mg/L (2.80–2.82 mmol/L) (reference range: 85–105 mg/L [2.12–2.62 mmol/L]) without symptomatic improvement. Surgery was proposed as the standard treatment, but the patient strongly declined due to anxiety about fetal risk. Given the urgency, her refusal of surgery, and favorable anatomical factors, we proposed a minimally invasive option: microwave ablation (MWA).

### Procedure

The patient was placed in a supine position with mild neck extension. An experienced anesthesiologist was present for continuous monitoring. During the ablation phase, the patient was asked to repeatedly count out loud to allow real-time assessment of voice integrity and early detection of any recurrent laryngeal nerve irritation.

Under local anesthesia with 2% lidocaine, hydrodissection using chilled 5% dextrose in water (D5W) was performed to create a > 5 mm safety margin between the adenoma and critical structures (esophagus, common carotid artery, and presumed recurrent laryngeal nerve path) (Figure 1E).

A 17-gauge, 10 cm microwave antenna with a 3.5 mm active tip was used. The antenna was introduced laterally to avoid traversing the thyroid. Ablation was initiated at the feeding artery of the adenoma to minimize intratumoral bleeding, followed by targeted zone-by-zone ablation. The entire procedure was performed under continuous ultrasound guidance. Power output was set at 25 watts throughout the procedure. Total procedure time was 23 min, with an ablation time of 3 min. Hydrodissection was repeated several times due to the adenoma’s volume, using a total of 120 mL of injected fluid.

Technical difficulty was increased due to the coexisting thyroiditis, which reduced echogenic contrast between the hypoechoic adenoma and thyroid tissue (Figures 1A,B). However, post-procedural imaging confirmed marked hypoechogenicity and complete loss of vascularity of the ablated lesion (Figures 1F,G).

The patient remained under observation for 1 h with neck cooling and was discharged without discomfort or complications.

## Results

**Figure 2:**
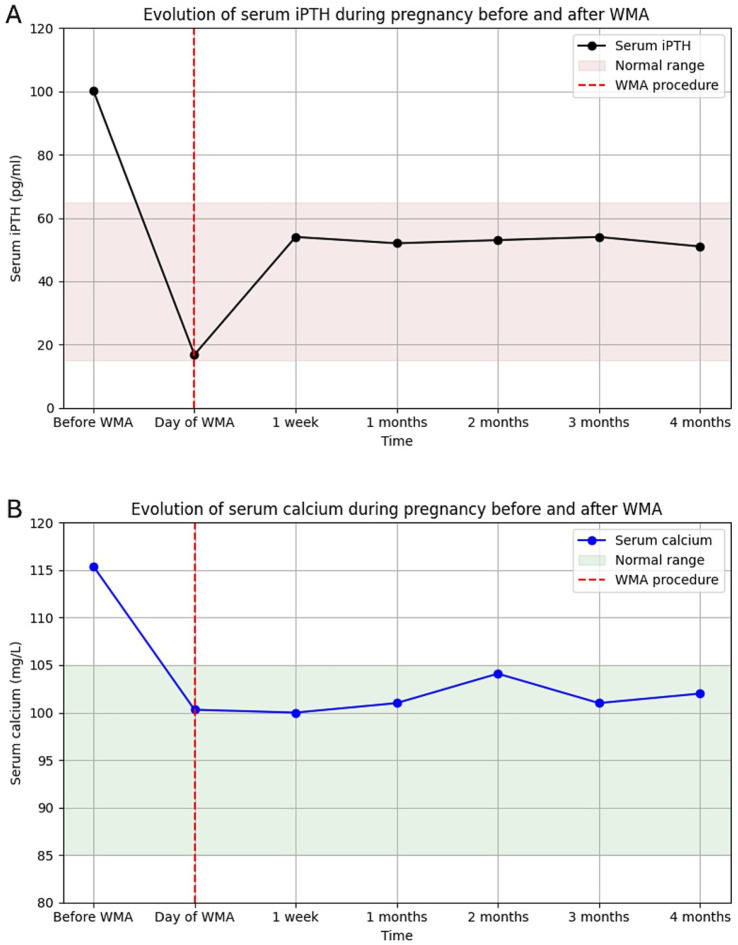
**(A)** Evolution of intact PTH and **(B)** corrected serum calcium from diagnosis to delivery after microwave ablation. Both normalized rapidly and remained stable.

One hour after completion of the procedure, iPTH dropped markedly to 16.8 pg./mL (15–65) (Figure 2A), with a concomitant normalization of serum calcium at 100.3 mg/L (2.51 mmol/L) (Figure 2B). One week later, iPTH had slightly increased but remained within the normal range at 54 pg./mL, while serum calcium remained stable at 100 mg/L (2.50 mmol/L). One month later, both PTH (51–54 pg./mL) and serum calcium (~100 mg/L [2.50 mmol/L]) remained stable, and this biochemical normalization persisted throughout the subsequent follow-up until delivery (Figure 2).

Urinary calcium also showed a considerable decrease after ablation and, when measured once during the third trimester, remained at the upper limit of normal for gestational age (401 mg/24 h).

Clinically, symptoms improved by the evening of the procedure and resolved completely within 3 days. Obstetric follow-up the following day confirmed a healthy fetus.

The remainder of the pregnancy progressed smoothly without complications. At 38 weeks of gestation, the patient gave birth by cesarean section to a healthy baby boy. Although neonatal follow-up data are not available in this report, postpartum maternal and neonatal monitoring is recommended in cases of maternal primary hyperparathyroidism due to potential complications.

## Discussion

Primary hyperparathyroidism (PHPT) during pregnancy is uncommon and often underdiagnosed, yet it can have significant maternal and fetal consequences, particularly when hypercalcemia is marked (1–3). Maternal complications include nephrolithiasis, pancreatitis, hyperemesis gravidarum, preeclampsia, and hypercalcemic crisis, while fetal complications range from intrauterine growth restriction to miscarriage, preterm delivery, and stillbirth (4–7). Early recognition and appropriate management are therefore crucial.

Management of PHPT in pregnancy is challenging. Conservative measures, such as hydration and a low-calcium diet, are often insufficient, while pharmacologic options such as calcitonin or cinacalcet have limited safety data in pregnancy (8–12, 16–18). Parathyroidectomy remains the standard of care and is generally recommended in the second trimester, when the risk to both mother and fetus is lowest (13, 14). In some urgent cases, such as acute pancreatitis related to primary hyperparathyroidism, surgery may be required outside this optimal period (9). However, surgery is not always feasible due to gestational timing, comorbidities, or patient preference.

Minimally invasive techniques such as thermal ablation have emerged as alternatives in selected patients. Both radiofrequency ablation and microwave ablation (MWA) have shown efficacy for parathyroid adenomas in nonpregnant populations (15, 19–24). Reports of their use in pregnancy are extremely scarce, with only one prior case of MWA for PHPT during pregnancy published (15). Our case adds to this limited evidence, demonstrating that MWA can achieve rapid and sustained normalization of calcium and PTH levels while being well tolerated and safe for the fetus.

In our patient, biochemical normalization occurred within hours and was maintained throughout pregnancy. Symptoms improved quickly, and obstetric follow-up confirmed healthy fetal development. The outcome was favorable, with the delivery of a healthy infant at 38 weeks. This observation suggests that MWA may represent a valuable therapeutic option in highly selected pregnant women when surgery is not possible, although further studies are needed to confirm its safety and efficacy.

This case presented specific clinical considerations due to the patient’s age (38 years), history of multiple miscarriages, and lack of living children. Given these factors and the ongoing pregnancy, careful consideration of maternal and fetal safety was required. A minimally invasive approach under local anesthesia was chosen to manage hypercalcemia while minimizing risks to the fetus.

### Limitations

This report has several limitations. It describes a single case with a relatively short follow-up, which limits the generalizability of the findings. Genetic testing for multiple endocrine neoplasia could not be performed due to resource constraints. Neonatal data were also limited, and the procedure was conducted by a single experienced operator. Therefore, while the outcome was favorable, further studies and longer follow-up are required to confirm the safety and reproducibility of microwave ablation for parathyroid adenomas during pregnancy.

## Conclusion

This case illustrates that microwave ablation may represent a feasible minimally invasive parties option for managing primary hyperparathyroidism during pregnancy in highly selected situations where surgery is contraindicated or declined. The procedure achieved rapid biochemical normalization, symptom relief, and favorable maternal and fetal outcomes in this individual case. However, given the exceptional nature of such situations and the lack of long-term follow-up data, no general conclusions can be drawn regarding its routine use. Further accumulation of clinical experience and multicenter collaboration are needed to better define the safety profile, efficacy, and potential indications of thermal ablation in this rare and challenging context.

Learning points:

- Primary hyperparathyroidism in pregnancy, although rare, can lead to significant maternal and fetal morbidity if not promptly recognized and managed.
- Parathyroidectomy in the second trimester remains the standard treatment, but it may not always be possible due to gestational timing, comorbidities, or patient preference.
- Minimally invasive techniques, such as microwave ablation, may represent a safe and effective alternative in highly selected cases.
- Individualized decision-making is essential, especially in precious pregnancies, where both maternal and fetal safety must be carefully balanced.

## Data Availability

The original contributions presented in the study are included in the article/supplementary material, further inquiries can be directed to the corresponding author.
